# Life-history strategy defends against disease and may select against physiological resistance

**DOI:** 10.1002/ece3.583

**Published:** 2013-05-08

**Authors:** Amanda K Gibson, Elsa Petit, Jorge Mena-Ali, Bengt Oxelman, Michael E Hood

**Affiliations:** 1 Department of Biology, Amherst CollegeAmherst, Massachusetts; 2 Department of Biology, Indiana UniversityBloomington, Indiana; 3 Department of Biology, Franklin & Marshall CollegeLancaster, Pennsylvania; 4 Department of Biology and Environmental Sciences, University of GothenburgGothenburg, Sweden

**Keywords:** Annual, disease resistance, *Microbotryum*, perennial, *Silene*

## Abstract

Host ecological traits may limit exposure to infectious disease, thereby generating the wide variation in disease incidence observed between host populations or species. The exclusion of disease by ecological traits may then allow selection to act against physiological defenses when they are costly to maintain in the absence of disease. This study investigates ecological resistance in the *Silene-Microbotryum* pathosystem. An estimated 80% of perennial *Silene* species host the anther-smut disease while no annuals harbor the disease in nature. Artificial inoculations of annual and perennial *Silene* plants, obtained from both natural and horticultural populations, demonstrate that the absence of disease in annuals is not explained by elevated physiological resistance. The annual habit is thus a powerful form of ecological defense against anther smut. Moreover, the higher susceptibility of annual species to anther smut relative to perennials supports the hypothesis of a loss of costly physiological resistance under ecological protection. The observation in annuals that physiological susceptibility is correlated with lower rates of flowering (i.e., lower fitness) suggests that variation in physiological resistance is costly in the absence of disease, even in a naїve *Silene* species. The absence of disease in natural populations of annuals combined with their high physiological susceptibility attest to the strength of host ecology in shaping the distribution of disease and to the dynamic nature of disease resistance.

## Introduction

Members of the same or closely related species frequently exhibit great disparities in their incidence of infectious disease. Explanations for this variation in susceptibility between individuals or populations are manifold, ranging from fine-scale variation in immune traits (Graham et al. 2010; Telfer et al. 2010) and social interactions (Tinsley 1989; Ezenwa 2004; Hawley et al. 2006) to broad-scale variation in environmental factors (Pedersen and Grieves 2008; Duffy et al. 2012) and demography (Nunn et al. 2003; Altizer et al. 2007; Lindenfors et al. 2007). Most of these host traits influencing pathogen susceptibility can be divided into two broad categories: physiology and ecology. Physiological defenses are biochemical, immunological, and structural mechanisms that determine the proportion of individuals remaining healthy upon direct exposure to a pathogen. Alternatively, ecological defenses encompass behavioral, life-history, and demographic traits that limit exposure to a pathogen (Ouborg et al. 2000).

Ecological differences have been cited in a wide variety of taxa to explain disease distributions or to estimate risk from emerging infectious diseases (e.g., Pedersen et al. 2007; Jones et al. 2008; Duffy et al. 2012; Johnson et al. 2012; Swinfield et al. 2012). Examples include age (Lafferty 1993; Jokela and Lively 1995; Fredensborg and Poulin 2006) or sex-specific behaviors (Alexander 1989; Ezenwa 2004), as seen in the North American desert toad: females spend less time in water and therefore carry only a quarter of the number of parasitic aquatic trematodes as do males (Tinsley 1989). Prevention of disease by large-scale demographic factors has been reported in primates (Nunn et al. 2003; Altizer et al. 2007) and carnivores (Lindenfors et al. 2007). In both cases, fewer parasites are found in species exhibiting reduced population density and connectivity. Similarly, Plowright et al. (2011) demonstrate that variation between populations of flying fox species in these same factors influences the size and persistence of Hendra virus outbreaks, as well as its probability for emergence in humans. These examples highlight the disparity in disease distributions that can exist within and between species when strong ecological contrasts are present.

Hood et al. (2010) recently surveyed over 40,000 herbarium specimens for the presence of the disease anther smut. All specimens were collected from natural populations of plants in the genus *Silene* and allied genera of the family Caryophyllaceae. This study revealed that the fungal disease is entirely restricted to perennial species. The contrast between annuals and perennials remains even after controlling for phylogenetic relationships within the clade. These results agree with those of Thrall et al. (1993), who found fewer literature reports of anther smut on annual than perennial species. Both studies attribute the lack of anther smut in annuals to the particular life cycle of the pathogen.

**Figure 1:**
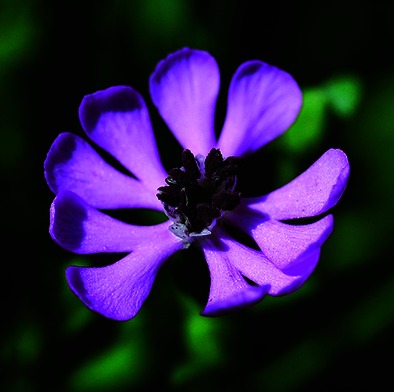
Expression of anther-smut disease upon inoculation of the annual plant species *Silene colorata* with the fungal pathogen *Microbotryum lychnidis-dioicae*. The pollen has been replaced by dark-colored fungal spores.

This disease results from infection by pollinator-transmitted fungal pathogens in the genus *Microbotryum*. Infection leads to host sterilization by abortion of female structures and replacement of pollen with dark-colored fungal spores (Fig. 1). Disease transmission occurs primarily through spores vectored by pollinators from infected to healthy plants (Alexander and Maltby 1990). Spore germination, meiosis, and mating of haploid sporidial cells of opposite mating types occur on the host plant surface prior to infection (reviewed in Giraud et al. 2008). The pathogens are believed to lack any environmentally-resilient resting stages, thus requiring overwintering within the living host. It is this feature of anther smut that has been hypothesized to make annual plants, which do not overwinter, inviable hosts of anther-smut disease (Hood et al. 2010). Accordingly, these studies suggested that the annual habit can function in the *Silene*-*Microbotryum* system as an ecological defense, restricting the disease's distribution.

Effects of other ecological traits have also been characterized in this system. For instance, the disease is absent from species in the Caryophyllaceae that have cleistogamous (i.e., “closed mating”) flowers, presumably due to lack of interaction with pollinators that vector the fungal spores (Thrall et al. 1993). Furthermore, despite exhibiting the perennial life history, *Silene* species threatened with extinction appear to have lost the disease, potentially due to their occurrence in small, fragmented populations where transmission is suppressed (Gibson et al. 2010). At the within-species level, *Silene latifolia* shows lower infection prevalence in genotypes with shortened or delayed flowering seasons (Alexander et al. 1993; Biere and Antonovics 1996).

The marked absence of disease on annual species is consistent with ecological resistance. The possibility remains, however, that the absence of anther smut in annual Caryophyllaceous species is instead due to strong physiological resistance, as prior studies on disease and life history were correlative (Thrall et al. 1993; Hood et al. 2010). The *Silene-Microbotryum* system is well suited to test the relative contributions of ecological and physiological resistance. Artificial inoculations of host plants with *Microbotryum* isolates allow direct, experimental estimation of physiological resistance to anther-smut disease. A genetic basis for resistance to anther smut has been demonstrated in multiple *Silene* species (Alexander 1989; Alexander et al. 1993; Cafuir et al. 2007). Moreover, it is one of the few natural systems where the costs of resistance have been quantified (Alexander 1989; Biere and Antonovics 1996).

In this study, we aim to directly measure physiological resistance of annuals to determine whether host ecological traits are responsible for their disease-free status. In addition, we assess the possibility that, in the presence of a strong ecological defense, natural selection may favor the loss of mechanisms of physiological resistance, which are often costly to maintain in the absence of disease (Alexander 1989; Simms 1992; Biere and Antonovics 1996). We would predict such selection to manifest as annual species exhibiting greater physiological susceptibility than perennial species. We estimate physiological resistance using experimental inoculations of annual and perennials in the genus *Silene* to compare infection rates upon direct exposure to *Microbotryum* pathogens. Moreover, we quantify the costs associated with variation in physiological resistance in one species of annual host. By addressing fundamental life-history differences as ecological determinants of disease risk, this study finds that host ecology governs pathogen distributions in nature and shapes the dynamics of host-pathogen coevolution.

## Material and Methods

### Model system

Fungal pathogens of the *Microbotryum violaceum* species complex (Basidiomycetes: Microbotryales) represent a suite of divergent, host-specific species (Le Gac et al. 2007; Lutz et al. 2008). Host species are primarily members of the Caryophyllaceae family and are particularly overrepresented in the *Silene* genus. Recently, Hood et al. (2010) estimated that approximately 80% of perennial species in the tribe *Sileneae*, within the Caryophyllaceae, are likely to host *Microbotryum* pathogens in nature. Infection causes anther-smut disease, of which the primary symptom is sterilization. Sterilization is often partial in the first season of infection, but anther smut is fully expressed in all flowers in subsequent years (Fig. 1). Effects upon host mortality are minimal, but the disease can impact population growth rates and persistence (Antonovics 2004).

### Artificial inoculations

All plant species used in this study are members of the tribe *Sileneae* in the Caryophyllaceae (Oxelman et al. 2001). Sources of seeds are provided in Table S1. A total of eight annual species, represented by nine populations, and five perennial species, represented by eight populations, were inoculated. Annuals were identified by a review of published species descriptions in floras (see Hood et al. 2010). The included species represent at least three independent origins of the annual habit in the *Silene* genus (Oxelman et al. 2001; Rautenberg et al. 2012, B. Oxelman, unpubl. data). Perennial hosts used as controls are species known to maintain anther smut in the wild. They were therefore predicted to be susceptible to disease under artificial inoculation. Controls were intended to verify efficacy of the inoculation technique and to provide standard disease rates for comparison to those of annuals.

Seeds were surface-sterilized prior to germination, and upon emergence of cotyledons, seedlings were inoculated with four μL of *Microbotryum* according to Hood and Antonovics (2000). Two different inoculation treatments were applied to each set of seeds. This approach was taken to increase the probability of inoculating annual species with compatible pathogen strains. Unlike perennial *Silene*, annual species are not infected in nature and therefore do not have a native strain of *Microbotryum*. The first treatment (the ‘combined’ inoculum) comprised a pooled mixture of a_1_ and a_2_ haploid cells (sporidial cultures) from field collections of *Microbotryum* isolates infecting 13 different host species and representing at least nine different pathogen species (Table S2) (Le Gac et al. 2007). The second treatment (the ‘single’ inoculum) comprised a mix of a_1_ and a_2_ sporidia from a single pathogen species, which was selected from the previous list (Table S2) in an attempt to minimize the genetic distance of the pathogen's native host (i.e., the host-of-origin) and the host used as the target of artificial inoculations (see ‘Estimating phylogenetic constraints’ below).

Plants were grown in the greenhouse until flowering, at which time they were assessed for the presence of *Microbotryum* spores in their anthers. Flowers were collected prior to opening, when petals had extended further than the calyx teeth, or immediately upon opening to minimize contamination between inoculated plants. Diseased flowers were scored for the normality of disease expression, including whether anther filaments were fully elongated and the anther sacks had ruptured to reveal powdery and dehiscent fungal spores. Flowers of all diseased individuals were collected and stored under desiccation.

If examination under the stereo microscope was insufficient to determine disease status, the presence of *Microbotryum* was ascertained using PCR amplification. DNA was extracted from anthers by the Chelex method described in Bucheli et al. (2001). Standard PCR reactions were performed using *Microbotryum*-specific primers that amplify variable regions of the internal transcribed spacer (ITS) region of the nuclear rRNA genes described in Hood et al. (2010). Presence of infection was assigned upon visual examination of PCR products after separation in 1% agarose gels.

### Estimating phylogenetic constraints

To investigate the potential influence of host specificity on pathogen success, the rates of infection and the normality of symptoms in the single inoculum treatment were tested for dependence upon the genetic distance between the inoculated annual species and the pathogen's perennial host-of-origin. Sequences for the internal transcribed spacer (ITS) region of the nuclear rRNA genes and the chloroplast *rps16* intron were obtained from the NCBI database, from the online *Sileneae* database (http://www.sileneae.info), or directly from plants in the greenhouse. Greenhouse specimens used for DNA sequencing were preserved as herbarium sheets. DNA was extracted using the Plant DNeasy Kit (Qiagen, Hilden, Germany) following the manufacturer's protocol. The nuclear ITS1-4 and plastid intron *rps16* regions were amplified from extracted plant DNA using standard PCR methods and primers given in White et al. (1990) and Popp and Oxelman (2007), respectively. Sequences were generated at the Molecular Genetics Core Facility at Penn State (http://med.psu.edu/web/core/molecular-genetics-services). GenBank (NCBI) accessions generated in this study are as follows: JX560218^ITS^ and JX560215^*rps16*^ for *S. cisplatensis*, JX560219^ITS^ and JX560216^*rps16*^ for *S. colorata*, JX560214^*rps16*^ for *S. gallica*, JX560220^ITS^ and JX560217^*rps16*^ for *S. germana*, and JX560221^ITS^ for *S. macrodonta*. Sequences for each locus were aligned using CLUSTAL-W implemented in MEGA4 (Tamura et al. 2007) and concatenated for analysis of genetic distances using the Kimura 2-parameter model with pairwise deletion.

### Quantification of costs of physiological resistance

Physiological resistance to *Microbotryum* can be costly, as demonstrated by a negative association with surrogate fitness measures in natural perennial hosts (Biere and Antonovics 1996; Biere and Honders 1996). To evaluate whether costs of physiological resistance are also present in naïve annual species, five families of the annual species *S. macrodonta* were used. The families were produced by self-pollinating plants grown from field-collected seeds. For each family, two sets of 60 plants were assessed for infection following separate inoculations with two phylogenetically distant *Microbotryum* species: *Microbotryum* originating from *S. latifolia* and *Microbotryum* from *Lychnis flos cuculi* (*N* surviving plants among families: 36 ± 4). For each family, two additional sets of plants were grown for two months without pathogen exposure to assess the proportion of plants that flowered as a measure of fitness (*N* among families: 15 ± 2).

### Statistical analysis

To assess differences among treatments in rates of infection and the normality of symptom development, statistical comparisons were made using generalized linear models in SPSS version 16 (SPSS Inc., Chicago, IL). A binomial logit function was assumed, and the model included the effect of host lifespan (annual vs. perennial), inoculum type (combined vs. single), and the interactions between these terms in predicting the dependent variable of the proportions of plants that became diseased. The analysis was performed on the complete dataset, including entries for each population nested within species (i.e., for *S. latifolia* and *S. noctiflora*) as well as with the plants from separate populations pooled within species. Regression analyses incorporating host phylogenetic distances were performed in SPSS using a weighted least-squares linear regression model, in which the frequency (*p*) of disease or of normal anther-smut symptoms was weighted by the inverse of its variance, where var(*p*) = *p*(1−*p*)/*n*. Data for perennial hosts, which received their endemic pathogen in the single inoculum treatment, were excluded from this analysis. Variations in infection rates and flowering patterns were analyzed with a full factorial general linear model in SPSS, with infection rate as the dependent variable and pathogen type and proportion flowering as the fixed factor and covariate, respectively. Proportional data were arcsine-square root transformed for analysis.

## Results

**Figure 2:**
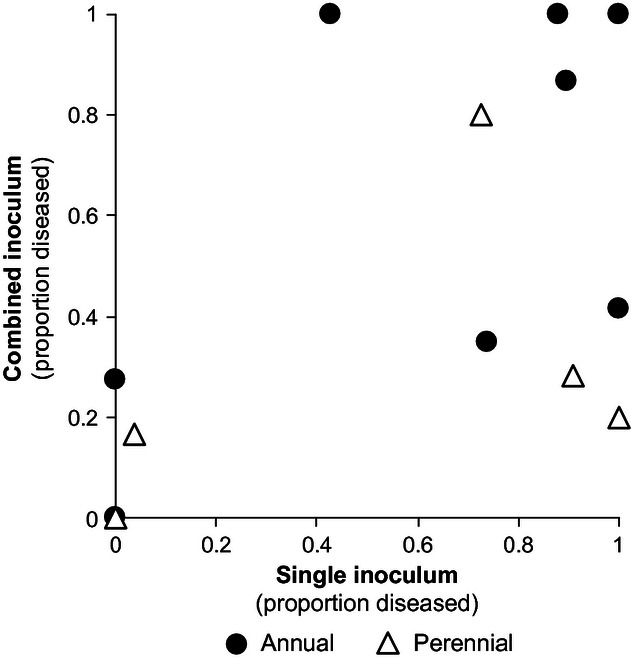
The relationship between the proportions of annual and perennial species infected as the result of inoculation with either a single or a combined mixture of *Microbotryum* species. Proportion of diseased individuals is given for five perennial (open triangles) and eight annual (closed circles) *Silene* species. Plants from multiple populations were pooled within species as indicated in the Material and Methods section, and sample size for the calculated proportions are shown in Table 1.

Four of five perennial species were successfully infected with *Microbotryum*. For three of these infected species, average infection rates across both inoculum treatments were greater than 60% (Table 1; Fig. 2). Seven of eight annual species were successfully infected with *Microbotryum*. For six of these infected species, average infection rates across both inoculation treatments were approximately 60% or greater. For two annual species, *S. cisplatensis* and *S. macrodonta*, average infection rates exceeded 90%.

**Table 1:** Proportion of annual and perennial host species diseased and displaying normal symptoms following inoculation with *Microbotryum* under single and combined treatments

	Proportion diseased (*N*)		Proportion normal disease expression (*N*)		
	
	Combined inoculum	Single inoculum	Combined inoculum	Single inoculum	Host source for single inoculum
**Annual species**					
*Atocion armeria*	0.87 (15)	0.89 (19)	0.93 (13)	0.89 (17)	*A. rupestre*
*Silene cisplatensis*	1.00 (11)	1.00 (10)	0.00 (11)	0.00 (10)	*V. alpina*
*Silene colorata*	0.27 (11)	0.00 (14)	0.67 (3)		*S. acaulis*
*Silene conica*	1.00 (7)	0.43 (7)	0.71 (7)	0.86 (3)	*S. latifolia*
*Silene gallica*	0.00 (14)	0.00 (14)			*S. acaulis*
*Silene germana*	0.42 (12)	1.00 (14)	0.00 (5)	0.00 (14)	*S. latifolia*
*Silene macrodonta*	1.00 (28)	0.88 (42)	1.00 (28)	0.95 (37)	*S. latifolia*
*Silene noctiflora*	0.42 (19)	0.66 (53)	0.13 (8)	0.40 (35)	*S. latifolia*
*Silene noctiflora*	0.30 (30)	0.92 (24)	0.13 (9)	0.73 (22)	*S. latifolia*
**Perennial species**					
*Atocion rupestre*	0.80 (10)	0.73 (11)	0.80 (8)	1.00 (8)	
*Silene italica*	0.20 (5)	1.00 (7)	1.00 (1)	1.00 (7)	
*Silene latifolia*	0.08 (29)	0.81 (36)	1.00 (12)	1.00 (36)	
*Silene latifolia*	0.38 (19)	0.85 (36)	1.00 (5)	1.00 (33)	
*Silene latifolia*	0.26 (24)	0.92 (21)	1.00 (2)	1.00 (17)	
*Silene latifolia*	0.41 (13)	1.00 (26)	1.00 (5)	1.00 (22)	
*Silene uniflora*	0.00 (47)	0.00 (47)			
*Silene vulgaris*	0.17 (36)	0.04 (26)	1.00 (6)	1.00 (1)	

Each proportion is followed by its sample size in parentheses. The host species from which the single inoculum was derived is given for each annual species. Each perennial species was singly inoculated with its native pathogen. The pathogen species associated with each host species are provided in Table S2.

When plant populations were pooled according to species, all model parameters significantly affected infection rates, including effects of annual versus perennial lifespan (Wald's χ^2^ = 5.3, df = 1, *P* = 0.021), inoculum treatments of a single *Microbotryum* species versus the combined mixture of multiple *Microbotryum* species (Wald's χ^2^ = 53.49, df = 1, *P* < 0.001), and the interaction between these terms (Wald's χ^2^ = 11.82, df = 1, *P* = 0.001). Overall, annual species became diseased at a higher rate than perennial species, and the combined inoculum treatment resulted in higher rates of infection. Additionally, annual species were more likely to become diseased from the combined inoculum treatment whereas perennial species were more likely to become diseased from the single inoculum treatment (Fig. 2). The analysis performed with the inclusion of separate populations within species did not alter the level of statistical significance for any model parameter.

Disease expression was not uniform across treatments. Infections with normal disease expression were considered to be those in which anthers of the infected host plant were fully developed and bore dehiscent spore masses. Perennial hosts displayed normal infection symptoms in 99.0% of diseased plants (*N* = 163), whereas annual species exhibited normal symptoms in only 59.6% of diseased plants (*N* = 222) (Table 1). These data were insufficient to run the full linear regression model to test for the interaction term (lifespan x inoculum type). The reduced model excluding the interaction term provided a highly significant main effect for lifespan (Wald's χ^2^ = 48.67, df = 1, *P* < 0.001), but no significant effect of inoculum type (Wald's χ^2^ = 0.86, df = 1, *P* = 0.355). The analysis performed with the inclusion of separate populations within species did not alter the level of statistical significance for any model parameter, noting that the separate populations within species had the same proportions of plants showing normal disease expression (Table 1).

Excluding data on perennial species, which received their endemic pathogens as inoculum, there was no significant evidence that specialization by the pathogen influenced infection rates among annual species, as would be determined by a negative relationship between infection rate and the genetic distance between the inoculated annual species and the pathogens' perennial hosts-of-origin (β = −0.392, *P* = 0.337). However, the genetic distance effect was significant in determining the proportion of infected plants with normal symptom development (β = −0.855, *P* = 0.030), with distant cross-inoculations more frequently resulting in underdeveloped anthers and/or nondehiscent spore masses.

**Figure 3:**
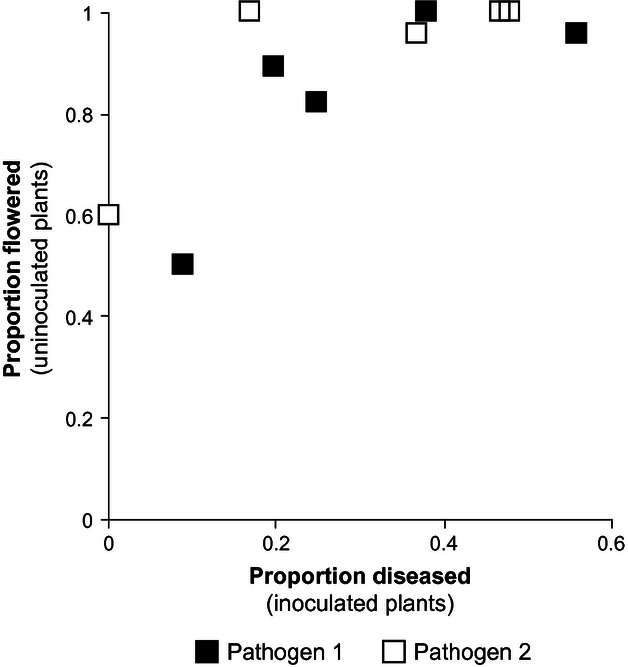
The relationship between the proportion infected by *Microbotryum* and flowering rate among families of the annual species *Silene macrodonta*. Infection rates were replicated across two pathogen species: Pathogen 1 = *Microbotryum* from *Lychnis flos cuculi* (closed squares) and Pathogen 2 = *Microbotryum* from *Silene latifolia* (open squares). Plant families were generated in the greenhouse. Infection rate was obtained using artificial inoculations while flowering rate was measured for uninoculated plants. Sample sizes for the calculated proportions are shown in Table S3.

Quantification of the costs of resistance in greenhouse-generated families of the annual species *S. macrodonta* identified a trade-off between resistance, when inoculated with *Microbotryum,* and flowering rate when uninoculated. A family's proportion of individuals that flowered among uninoculated plants was a significant positive predictor of infection rates among inoculated plants (transformed data, effect of flowering rate F(2, 1) = 17.07, *P* = 0.007) (Fig. 3). Effects of pathogen type and their interaction were not significant.

## Discussion

Anther-smut disease exclusively infects perennial plant species in nature, suggesting that an incompatibility with this fungal pathogen's disease cycle confers ecological protection in annual plant populations (Thrall et al. 1993; Hood et al. 2010). In this study, we compared the susceptibility to infection of annual and perennial *Silene* hosts in order to assess whether host ecology is a valid explanation for the absence of disease observed in annual species. We also investigated the potential loss of physiological resistance in annual hosts, as would be consistent with natural selection against costly physiological mechanisms of defense when there is strong ecological protection (Simms 1992). Our results indeed provide empirical support for the hypothesis that the annual habit serves as a form of ecological resistance against anther-smut disease. Moreover, they are consistent with a loss of physiological resistance under ecological resistance: annuals show lower physiological resistance than perennial species, and we demonstrate costs of physiological resistance in naïve annuals.

Attributing the absence of disease in annual populations to ecological rather than physiological resistance requires that artificial inoculations produce infection rates on annuals that are statistically as great as or greater than on perennials hosts. We provide evidence here that annual species become diseased at a significantly higher rate than perennial species under artificial inoculation. As verification of the artificial inoculation protocol used here, we find that infection rates on perennial species are comparable to those measured in prior studies using similar techniques (Biere and Honders 1996; Antonovics et al. 2002; Hood 2003; Gold et al. 2009). Moreover, the variation in infection rates observed between species is unlikely to be a technical artifact, as previous greenhouse and field experiments have shown that it represents heritable variation in physiological resistance (Alexander et al. 1993; Biere and Honders 1996). Therefore, our finding of high rates of infection upon direct exposure to *Microbotryum* supports the hypothesis that ecological factors are the primary determinants preventing disease persistence in natural populations of annual *Silene* species (Hood et al. 2010).

The annual habit of the *Silene* hosts addressed here contributes to disease protection in a manner similar to other life-history traits, such as lifespan and timing of reproductive maturity. Many studies implicate changes in life-history traits as adaptive responses to parasite pressure. If the threat of parasitism accumulates with age and parasitism is associated with a reduction in fecundity, life-history theory predicts a shift toward earlier host reproduction in the presence of parasites (Minchella 1985; Hochberg et al. 1992; Agnew et al. 2000). Similarly, earlier reproduction or shortened lifespan can, under certain conditions, reduce the need for an individual to invest in costly resistance mechanisms, such as innate immunity (Minchella 1985; Miller et al. 2007; Lee et al. 2008). Empirical studies have supported these predictions (as reviewed in Agnew et al. 2000). Agnew et al. (1999) observed accelerated development and earlier reproduction in infected female mosquitoes. Three separate studies of gastropods show a significant negative correlation between average size at reproductive maturity and trematode infection prevalence, consistent with reproduction at an earlier age in parasitized populations (Lafferty 1993; Jokela and Lively 1995; Fredensborg and Poulin 2006). Our finding that the annual habit is a form of ecological resistance against anther-smut disease (Thrall et al. 1993; Hood et al. 2010) further supports these previous studies in demonstrating that life-history traits serve as powerful defenses against parasitism.

A key distinction between the annual habit and the life-history shifts discussed in these previous studies must, however, be made: it cannot be inferred that the annual habit evolved as an adaptive response to parasitism. We show here that annual species are susceptible to infection with anther smut. Therefore, individual plants in a diseased population may not experience a gain in fitness by reducing lifespan. Rather, the primary benefit of the annual habit seems to manifest at the group level, with populations of annuals being incapable of maintaining the pathogen across years (Hood et al. 2010). Further studies are required to determine whether a group selection argument is required for disease to drive a shift from a perennial to annual life history. An alternative scenario is that the annual habit has evolved in response to unrelated selection pressures and has subsequently influenced disease transmission and infection. Indeed, transitions between the annual and perennial habit depend upon a complex balance of investment in reproduction and growth so as to optimize lifetime reproductive fitness. This balance is altered by numerous external factors, including environmental stability and, perhaps, disease (Cole 1954; Gadgil and Bossert 1970; Charnov and Schaffer 1973; Gaines et al. 1974; Hart 1977).

Regardless of the evolutionary history of the host trait, a mechanism that excludes a common and damaging disease can be expected to leave a distinct mark upon the patterns of natural selection in the host. We show here that the strong ecological resistance of annual *Silene* hosts is associated with lower physiological resistance: upon direct exposure, annuals became infected by *Microbotryum* more frequently than did perennial hosts. This result suggests that costly physiological resistance is lost under powerful ecological resistance. Indeed, we find evidence for such costs of physiological resistance in annuals: variation in susceptibility among families of the annual *S. macrodonta* was correlated with flowering patterns in a manner similar to reported costs of resistance in perennial hosts. Biere and Antonovics (1996) showed that resistant *S. latifolia* genotypes suffer a fitness cost relative to more susceptible genotypes in the absence of disease. Alexander et al. (1996) found that late-flowering genotypes of *S. latifolia* became less diseased in the field, and Biere and Honders (1996) suggested that physiological resistance was also associated, albeit indirectly, with delayed flowering. Our study suggests that a similar correlation between resistance and flowering may exist even in potential host species that have not experienced the disease historically. Preexisting variation in resistance can have major implications for the emergence of new diseases. Accordingly, studies on a broader range of annual plants are needed to assess the generality of this variation.

It is probable that our estimation of susceptibility here even underestimates the extent to which physiological resistance is absent in annual species. Annual species are disease free in nature and so were inoculated with nonendemic pathogens. In multiple studies, such novel host-pathogen combinations perform relatively poorly, resulting in lower infection rates than inoculations of *Microbotryum* strains on their hosts-of-origin (Sloan et al. 2008; de Vienne et al. 2009). A significant negative correlation was lacking between infection rate and genetic distance from the hosts-of-origin to inoculated annual species; this may reflect a lack of statistical power or an insufficient range of host genetic distances. However, the development of abnormal disease symptoms was elevated in annual hosts and was significantly affected by the genetic distance between hosts-of-origin and inoculated annual species. Abnormal symptoms have been observed in cross-species inoculations and host shifts and are interpreted as a result of host specialization by *Microbotryum* (Antonovics et al. 2002; Lopez-Villavicencio et al. 2005; Sloan et al. 2008). Therefore, the high infection rates obtained here on annuals exposed to maladapted pathogens indicate a potentially more significant lack of physiological resistance than that inferred from observed infection rates alone. A long-term evolutionary consequence of this loss of physiological resistance may be to limit transitions from the annual to perennial habit in *Silene*, given the threat of acquiring anther smut in the absence of physiological defenses. Transitions between habits are frequent in the evolutionary history of the *Silene* genus, but unfortunately little is currently known regarding the directions of these evolutionary events (Hood et al. 2010).

The physiological susceptibility of annuals may also have a short-term impact on community-level disease dynamics. Pathogen spread and persistence is influenced by the interaction of multiple host populations that differ in their tolerance and competency as hosts (Fenton and Pedersen 2005; Hall et al. 2009; Kelly et al. 2009). As discussed above, highly susceptible annual plants can contract anther smut from sympatric diseased perennials and become transmissive within their single growing season. The movement of spores between sympatric annuals and perennials could augment transmission in perennial populations that are otherwise unsuitable for pathogen persistence (due to, e.g., high fragmentation, small population size, or high frequency of physiological resistance) (Carlsson-Granér and Thrall 2002; Gibson et al. 2010). Further studies of this disease in regions of high *Silene* species richness, with a mixture of annual and perennial habits, should be undertaken to determine the potential spillover and spillback dynamics of this disease.

In summary, we have complemented the many studies that examine the interactions of host ecology and infectious disease with experiments that assess the effects of host life history upon physiological resistance. Our results demonstrate that addressing the combination of physiological and ecological disease resistance in an appropriate model system provides insights into the impact of a pathogen introduction on novel host environments. Moreover, the phenomenon of ecological disease resistance explored in this study contributes to describing the complex phylogenetic and geographic patterns of distribution of an infectious disease in nature.

## Supporting Information

**Table S1.** Identification of the *Silene* species used as annual and perennial hosts.

**Table S2.** Identification of *Microbotryum* species used in experimental inoculations.

**Table S3.** The relationship between the proportion infected by *Microbotryum* and flowering rate among families of the annual species *Silene macrodonta*.
