# Supplementary material for: Life-history strategy defends against disease and may select against physiological resistance
Source: Ecol Evol. 2013 May 8;3(6):1741–50. doi: 10.1002/ece3.583 (PMC3686206; doi:10.1002/ece3.583)
Supplement: Supplementary file 1 [file ece30003-1741-SD1.doc]

**Life-history strategy defends against disease and may select against physiological resistance.**

Amanda K. Gibson, Elsa Petit, Jorge Mena-Ali, Bengt Oxelman, and Michael E. Hood

Ecology and Evolution, 2013

**Supporting Information**

| **Table S1: Identification of the *Silene* species used as annual and perennial hosts**. Species name, the location of the natural population from which seeds were collected, and the collector of seeds used in experimental inoculations are given and grouped according to life-history strategy. | | | | | |
| --- | --- | --- | --- | --- | --- |
| **Annual Species** | **Locality** | | **Source/Collector** | | |
|
| *Atocion armeria* (L.) Raf. | | Virginia | | J. Antonovics | |
| *Silene cisplatensis* Cambess. | | Uruguay | | B. Oxleman | |
| *Silene colorata* Poir. | | Italy | | KPR* | |
| *Silene conica* L. | | Italy | | M. Maranella | |
| *Silene gallica* L. | | Uruguay | | B. Oxelman | |
| *Silene germana* J. Gay | | Spain | | S. Talavera | |
| *Silene macrodonta* Boiss. | | Turkey | | B. Oxelman | |
| *Silene noctiflora* L. | | Czech Republic | | KPR* | |
| *Silene noctiflora* L. | | Czech Republic | | KPR* | |
|  | |  | |  | |
| **Perennial Species** | | **Locality** | | **Source/Collector** | |
| *Atocion rupestre* (L.) Oxelman | | France | | T. Giraud | |
| *Silene italica* (L.) Pers. | | Italy | | M.E. Hood | |
| *Silene latifolia* Poir. | | Massachusetts | | M.E. Hood | |
| *Silene latifolia* Poir. | | France | | T. Giraud | |
| *Silene latifolia* Poir. | | Hungary | | KPR* | |
| *Silene latifolia* Poir. | | Slovakia | | KPR* | |
| *Silene uniflora* Roth | | United Kingdom | | M.E. Hood | |
| *Silene vulgaris* (Moench) Garcke | | Virginia | | J. Antonovics | |
| *KPR indicates the Gardeners' Club Slovakia <http://www.kpr.eu/en/> | | | | |  |

| **Table S2. Identification of *Microbotryum* species used in experimental inoculations.** *Microbotryum* species name, the host-of-origin’s species name, and the location of the natural population from which fungal material was collected are provided. References for each species designation are also indicated.   | ***Microbotryum* Species^1^** | **Host-of-origin** | **Collection Site** | | --- | --- | --- | | *M. lagerheimii sensu lato* | *Atocion rupestre* | Chambery, France | | *M. dianthorum* | *Dianthus carthusianorum* | San Baronto, Italy | | *M. lagerheimii sensu stricto* | *Viscaria alpina* | Kirkjubaejarklaustur, Iceland | | N/A^2^ (MvLfc)^3^ | *Lychnis flos-cuculi* | Great Cumbrae Island, UK | | *M. saponariae* | *Saponaria ocymoides* | Ch’ Mad’ Delle Grazie, Italy | | *M. silenes-acaulis* | *Silene acaulis* | Lac de Puy Vachier, France | | *M. aff. violaceum* | *Silene caroliniana* | Gilbert’s Creek, KY, USA | | *M. violaceo-verrucosum* | *Silene italica* | Barisciano, Italy | | *M. lychnidis-dioicae* | *Silene latifolia* | Lamole, Italy | | N/A | *Silene lemmonii* | Tall Creek Horse Camp, CA, USA | | N/A | *Silene notarisii* | Campo Imperatore, Italy | | *M. violaceum sensu stricto* | *Silene nutans* | Lac de Puy Vachier, France | | *M. violaceum sensu lato* | *Silene paradoxa* | Parc Nat. Gran Sasso, Italy | |
| --- | --- | --- | --- | --- | --- | --- | --- | --- | --- | --- | --- | --- | --- | --- | --- | --- | --- | --- | --- | --- | --- | --- | --- | --- | --- | --- | --- | --- | --- | --- | --- | --- | --- | --- | --- | --- | --- | --- | --- | --- | --- | --- |
|  |
| ^1^Species designations are given as in Abbate and Hood , Denchev *et al*. , Denchev *et al.*, Lutz *et al.* , Kemler *et al*. , and Piątek *et al*. . ^2^N/A indicates that no species designation is established. ^3^Le Gac *et al.* designations are given in parentheses as available. |

| **Table S3: The relationship between the proportion infected by *Microbotryum* and flowering rate among families of the annual species *Silene macrodonta*.** Infection rates were replicated across two pathogen species: Pathogen 1 = *Microbotryum* from *Lychnis flos cuculi* (closed squares) and Pathogen 2 = *Microbotryum* from *Silene latifolia* (open squares). Plant families were generated in the greenhouse. Infection rate was obtained using artificial inoculations while flowering rate was measured for uninoculated plants. Sample size for the calculated proportions are shown in parentheses. | | |
| --- | --- | --- |
|  | **Proportion disease*** | **Proportion flowering**** |
| **Family ID** | **Pathogen 1** |  |
| 2 | 0.09 (54) | 0.50 (16) |
| 6 | 0.56 (55) | 0.96 (24) |
| 7 | 0.2 (25) | 0.89 (10) |
| 8 | 0.38 (47) | 1.00 (17) |
| 10 | 0.25 (24) | 0.82 (11) |
|  |  |  |
| **Family ID** | **Pathogen 2** |  |
| 2 | 0 (30) | 0.60 (14) |
| 6 | 0.37 (46) | 0.96 (23) |
| 7 | 0.47 (17) | 1.00 (9) |
| 8 | 0.17 (35) | 1.00 (16) |
| 10 | 0.48 (23) | 1.00 (11) |
|  |  |  |
| * In parentheses are the total numbers of plants inoculated | | |
| ** In parenthesies are the total number of seedlings sown | | |
